# Draft Genome Sequence of *Mycobacterium colombiense*

**DOI:** 10.1128/genomeA.00119-17

**Published:** 2017-04-06

**Authors:** Amar Bouam, Catherine Robert, Anthony Levasseur, Michel Drancourt

**Affiliations:** Aix Marseille Univiversité, CNRS, IRD, INSERM, AP-HM, URMITE, IHU - Méditerranée Infection, Marseille, France

## Abstract

*Mycobacterium colombiense* is a rapidly growing mycobacterium initially isolated from the blood of an HIV-positive patient in Colombia. Its 5,854,893-bp draft genome exhibits a G+C content of 67.64%, 5,233 protein-coding genes, and 54 predicted RNA genes.

## GENOME ANNOUNCEMENT

*Mycobacterium colombiense* is an acid-fast, nonmotile, rod-shaped mycobacterium that grows in 3 weeks, producing rough, nonpigmented colonies. It was initially isolated from the blood of four HIV-coinfected patients in Colombia in 1995 (1). Further isolates have been made from diseased lymph nodes and respiratory and stool specimens (2–4), as well as from the skin biopsy of a 17-year-old boy with disseminated cutaneous infection (5). *M. colombiense* was also isolated from hospital water in dental units. To our knowledge, it has never been isolated from animals. We performed the whole-genome sequencing of *M. colombiense* CSURP297 in order to describe its genomic content and to determine its phylogenetic relationships for facilitating the detection and identification of this species.

*M. colombiense* CSURP297 (Collection de Souches de l’Unité des Rickettsies, Marseille, France) was cultured in MGIT Middlebrook liquid culture (Becton, Dickinson, Le Pont-de-Claix, France) at 37°C in a 5% CO_2_ atmosphere. *M. colombiense* CSURP297 genomic DNA was sequenced by Illumina MiSeq runs (Illumina Inc, San Diego, CA, USA) with the mate-pair strategy using the Nextera Mate Pair sample prep kit (Illumina). The index representation for *M. colombiense* CSURP297 was determined to be 14.21%. A total of 1,092,357 paired reads were filtered per the read qualities. These reads were trimmed using Trimmomatic (6) and then assembled into scaffolds using SPAdes version 3.5 (7, 8) before manual finishing. SSPACE version 2 (9) and Opera version 2 (10) were used to combine the contigs helped by GapFiller version 1.10 (11). This yielded a draft genome consisting of 14 scaffolds composed of 123 contigs, for a total of 5,854,893 bp and a G+C content of 67.64%. Noncoding genes and miscellaneous features were predicted using RNAmmer (12), ARAGORN (13), Rfam (14), PFAM (15), and Infernal (16). Coding DNA sequences (CDSs) were predicted using Prodigal (17), and functional annotation was achieved using BLASTp against the GenBank database (18) and the Clusters of Orthologous Groups (COG) database (19, 20). The genome was shown to encode 54 predicted RNAs, including one each of the 5S rRNA, 16S rRNA, and 23S rRNA genes and 51 tRNAs. A total of 3,923 genes (74.97%) were assigned a putative function; 64 genes were identified as ORFans (1.22%); and 1,088 genes (20.79%) were annotated as hypothetical proteins. The *M. colombiense* CSURP297 genome was further incorporated into *in silico* DNA-DNA hybridization (DDH) (21) with reference genomes selected based on 16S rRNA gene proximity; DDH values were estimated using the GGDC version 2.0 online tool (22). This analysis yielded 31.95% ± 3.46 similarity with *M. intracellulare* ATCC 13950, 30.45% ± 3.46 with *M. avium* 104, 23.15% ± 3.32 with *M. szulgai* strain ACS1160, 22.8% ± 3.39 with *M. haemophilum* DSM 44634, 22.6% ± 3.39 with *M. tuberculosis* H37Rv, 22.55% ± 3.32 with *M. caprae* strain Allgaeu, and 22.15% ± 3.32 with *M. marinum* M, confirming at the genome level the taxonomic assignment of *M. colombiense* into the *M. avium* complex.

### Accession number(s).

The *M. colombiense* CSURP297 genome sequence has been deposited at EMBL under the accession number FUEH00000000.
